# When the Lung Refuses to Expand: A Deep Dive Into a Pneumothorax Ex Vacuo Case

**DOI:** 10.7759/cureus.46565

**Published:** 2023-10-06

**Authors:** Mena Louis, Lucas Canaan, John Hastings, Navneeth Bongu, Hardeep Singh

**Affiliations:** 1 General Surgery, Northeast Georgia Medical Center Gainsville, Georgia, USA; 2 Surgery, Northeast Georgia Medical Center Gainsville, Georgia, USA; 3 Cardiothoracic Surgery, Northeast Georgia Medical Center Gainsville, Georgia, USA; 4 Pulmonary and Critical Care, Northeast Georgia Medical Center Gainsville, Georgia, USA; 5 Research, Northeast Georgia Medical Center Gainsville, Georgia, USA

**Keywords:** video-assisted thoracoscopic surgery (vats), intercostal nerve blockade., decortication, thoracotomy, cerebral palsy, pleural effusion, trapped lung, pneumothorax ex vacuo

## Abstract

Pneumothorax ex vacuo and trapped lung represent challenging clinical entities, especially in the context of pre-existing comorbidities. This case report outlines the diagnostic and management pathway of a 38-year-old patient with cerebral palsy who initially presented with empyema. Following the evacuation of the empyema, the patient developed pneumothorax ex vacuo, a rare phenomenon occurring due to a vacuum-like negative intrapleural pressure initiated by lung collapse.

Initially suspected to have an infectious etiology based on laboratory findings, the patient was later found to have a large hydropneumothorax through a combination of imaging, laboratory studies, and clinical evaluations without confirming infection or malignancy. Despite interventions including Tissue Plasminogen Activator (TPA) and Deoxyribonuclease (DNAse) administration to facilitate pleural drainage, the patient's condition persisted, necessitating a surgical intervention that evolved from a minimally invasive video-assisted thoracoscopic surgery (VATS) to a more invasive thoracotomy due to unforeseen pleural thickening.

The patient's pre-existing condition of cerebral palsy increased his susceptibility to respiratory complications, including empyema, due to the risk of aspiration. This case highlights the importance of a multidisciplinary approach in managing such complex clinical scenarios. It also serves as a clinical reminder that pneumothorax ex vacuo is generally benign and does not typically require chest tube placement, as the primary issue is an unexpandable lung that is unresponsive to pleural drainage.

The report emphasizes the need for flexible surgical planning and robust postoperative management to optimize patient outcomes. It also clarifies the distinct pathophysiology of pneumothorax ex vacuo compared to primary or secondary pneumothorax, advocating for a comprehensive diagnostic approach and the crucial role of a multidisciplinary team in the management of such intricate cases.

## Introduction

Pneumothorax ex vacuo, a phenomenon less frequently encountered in clinical practice, manifests when there is an inability of the lung to re-expand post drainage of a pleural effusion, leading to the presence of air in the pleural space [1]. This occurrence is not due to a breach in the visceral pleura, a characteristic that distinguishes it from primary or secondary pneumothorax, but rather due to the formation of a thick fibrous peel that entraps the lung, preventing its re-expansion [2,3].

In the backdrop of this clinical scenario, it is crucial to emphasize that the primary issue is not the pleural effusion itself. The unexpandable lung is the main concern, a condition often initiated by a lung collapse that induces a negative pressure in the pleura, gradually drawing fluid into this space [4].

This sets a stage where therapeutic thoracentesis can momentarily open a small aperture in the lung, allowing air to enter the pleural space and alleviate the “vacuum” effect, a procedure not replicated in diagnostic thoracentesis [1,4].

Despite its benign nature, which rarely progresses to a tension pneumothorax, and the general asymptomatic presentation in patients, it poses a diagnostic conundrum, steering clinicians away from the conventional treatment pathways. The rarity of this condition necessitates a deep delve into its intricacies, offering a rich learning ground for clinicians to understand and manage this unique presentation effectively.

In this case report, we navigate through the diagnostic and therapeutic journey of a 38-year-old patient with cerebral palsy who presented with empyema and later developed pneumothorax ex vacuo post-evacuation, shedding light on the critical considerations and adaptive strategies employed in managing such rare presentations, and emphasizing the benign nature of this disease.

## Case presentation

A 38-year-old patient with a history of cerebral palsy and chronic gait abnormalities presented to the Emergency Department with a three-week history of progressive dyspnea. The patient had no associated symptoms like fever, cough, or chest pain.

Upon admission, the patient exhibited sinus tachycardia with a heart rate of 124 beats per minute. Other vital signs remained within normal limits. Laboratory tests revealed signs of acute inflammation, including leukocytosis, elevated levels of C-reactive protein (CRP), and reactive thrombocytosis (Table 1). Arterial blood gases were normal. Broad-spectrum antibiotics were started empirically.

**Table 1:** Lab values on admission.

Lab value	Result	Ref Range & Units
Sodium	134 mmol/L	135 - 148 mmol/L
White Blood Cell Count (WBC)	31.3 K/uL	4.8 - 10.8 K/uL
Hemoglobin	8.5 g/dL	14.0 - 18.0 g/dL
Platelets	1,032 K/uL	130 - 400 K/uL
C-reactive protein	27.20 mg/dL	0.00 - 0.60 mg/dL

**Figure 1:**
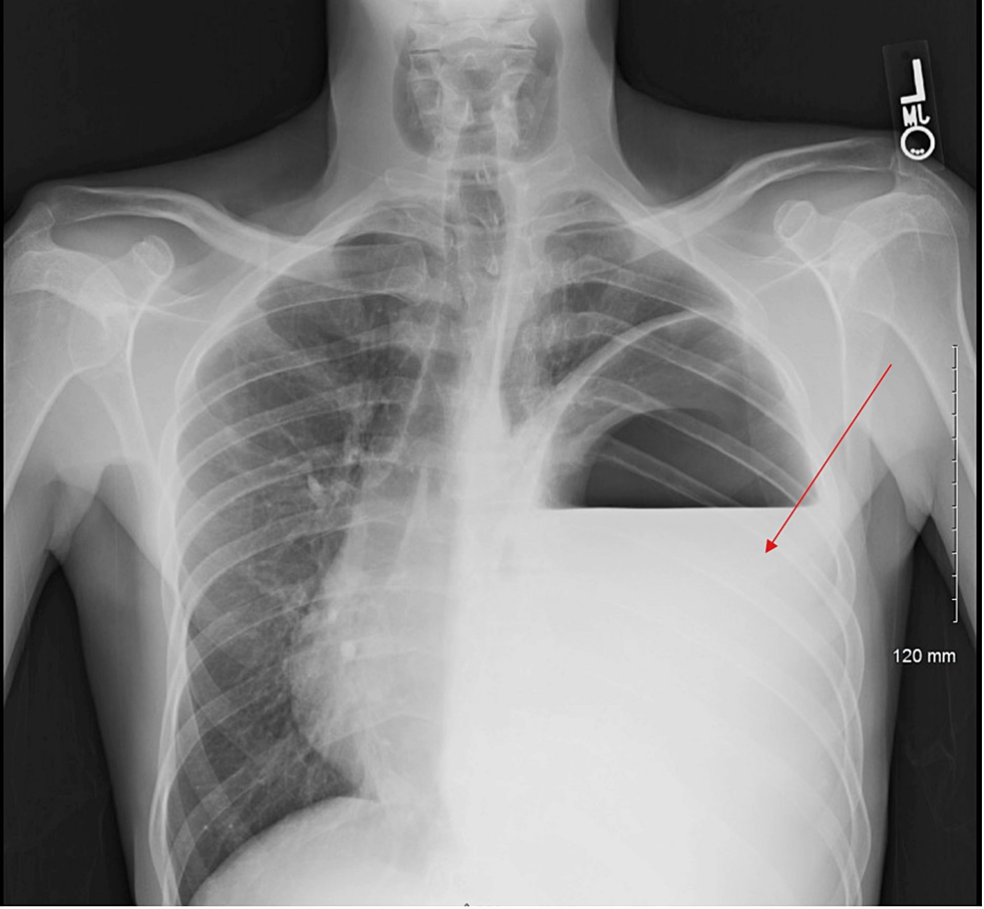
Chest X-ray image A significant air-fluid level (red arrow) is seen in the left hemithorax representing a large loculated pleural effusion/Hydro pneumothorax.

**Figure 2:**
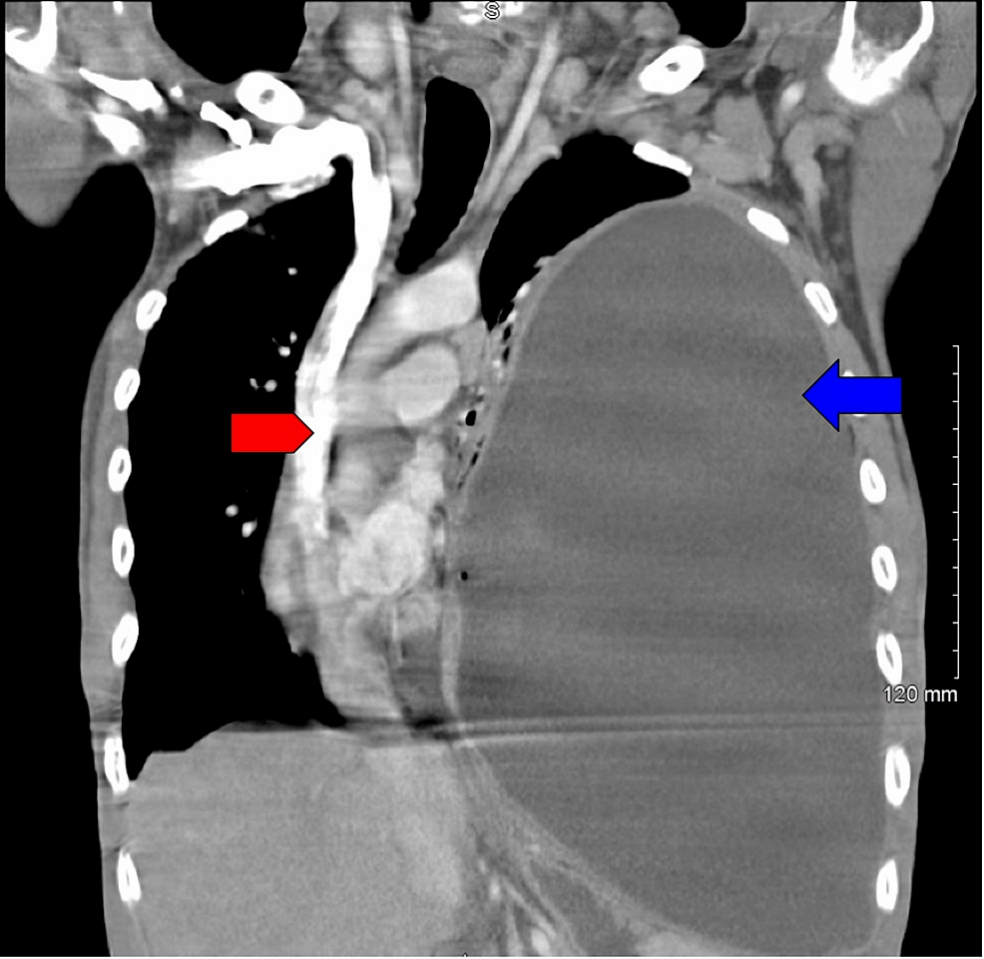
CT scan image (coronal) A coronal CT scan shows a considerable effusion of the left hemithorax (blue arrow) with significant mass effect upon the cardiac and mediastinal silhouette to the right with tracheal deviation (red pentagon).

**Figure 3:**
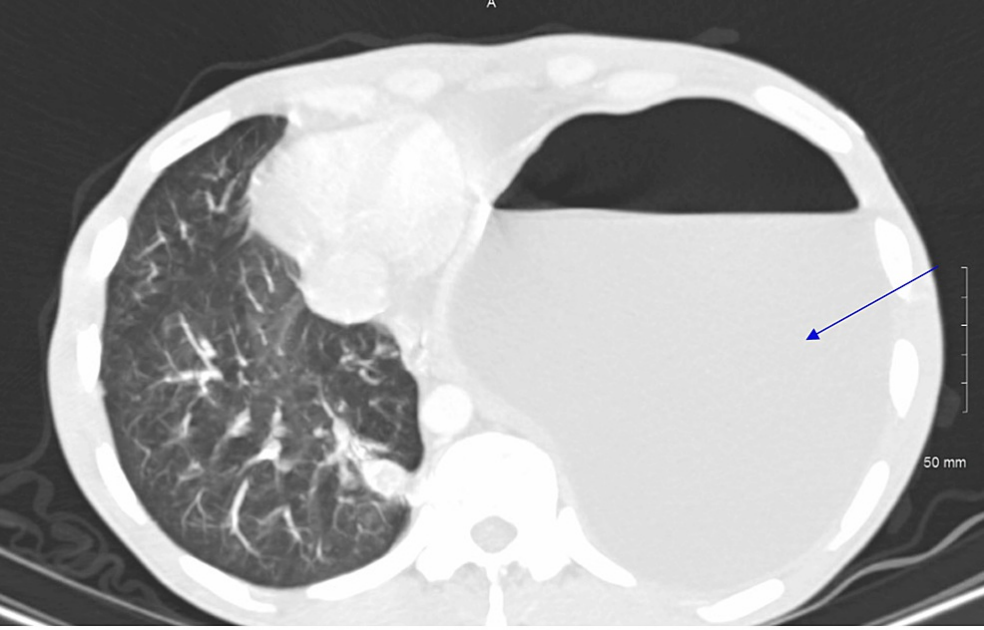
CT scan (axial) Axial CT scan shows a large air-fluid level (blue arrow) is seen in the left hemithorax with associated cardiac and mediastinal shift to the right.

A chest X-ray obtained in the Emergency Department revealed hydropneumothorax (Figure 1). This was followed by a CT scan of the chest which displayed a substantial effusion in the left hemithorax, causing a significant mediastinal rightward shift (Figures 2-3).

**Figure 4:**
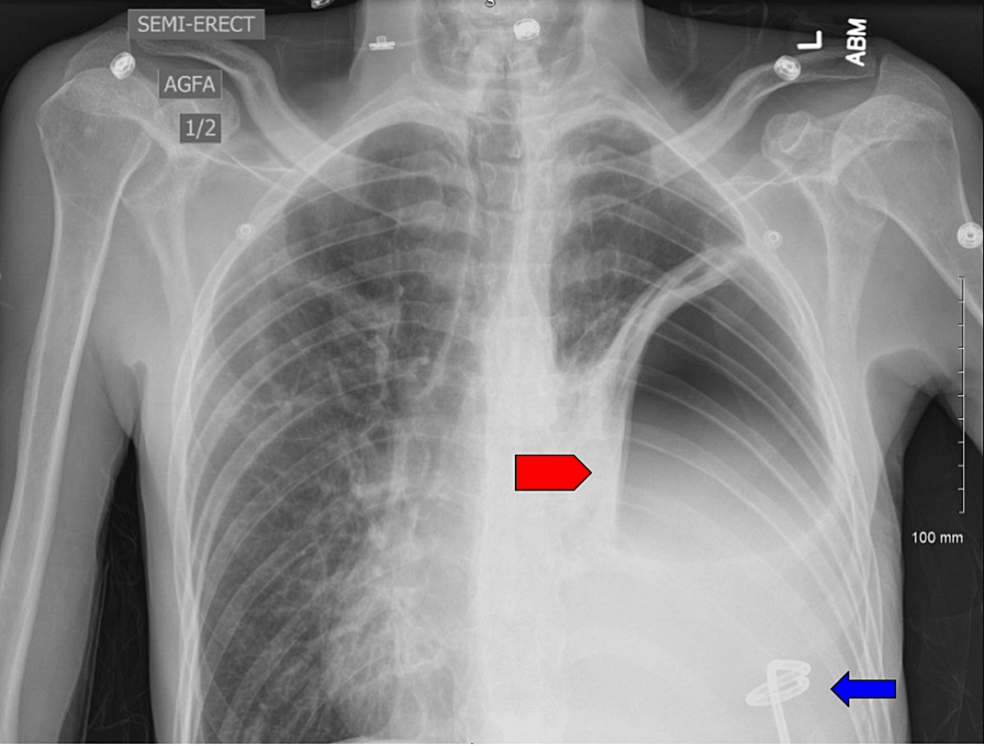
Chest X-ray on day 3 of admission Left lung base pleural catheter (blue arrow) with a loculated pneumothorax at the left lung base (red pentagon), reflecting pneumothorax ex vacuo.

Diagnostic interventions included an ultrasound-guided chest tube insertion, which initially drained 1100 cc of exudative fluid. Despite subsequent treatments with Tissue Plasminogen Activator (TPA) and Deoxyribonuclease (DNAse), imaging studies displayed a left-sided hydropneumothorax, adjacent compressive atelectasis, and increasing pulmonary vascular congestion. On day 3 of admission, a chest X-ray provided evidence of pneumothorax ex vacuo (Figure 4).

**Figure 5:**
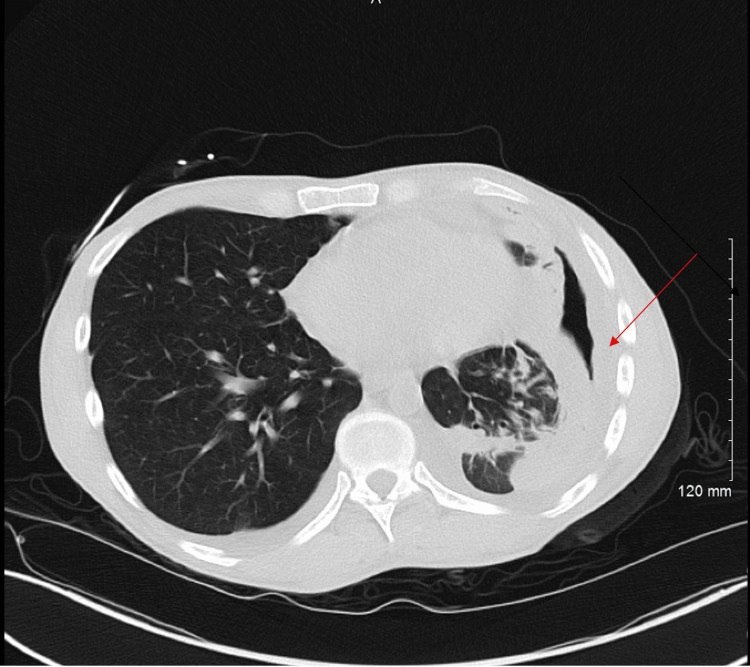
CT of the chest (axial) Axial CT of the chest shows hydropneumothorax on the left, with thickening of the visceral pleura (red arrow) is observed.

Pleural fluid was consistent with neutrophilic inflammation and mucus, which was consistent with empyema. Cultures and cytological analyses ruled out infection and malignancy. A chest CT scan was conducted on the fourth day of the hospital stay, revealing ongoing ex vacuo changes and pleural thickening, as illustrated in Figure 5.

Given the failed re-expansion of the left lower lobe and pleural thickening on the CT scan, the decision was made to proceed with surgical decortication. Initially, the approach involved Video-Assisted Thoracoscopic Surgery (VATS), but due to extensive pleural thickening and significant adhesions, conversion to an open left posterior lateral thoracotomy was required. Despite encountering challenging adhesions, maximum decortication was performed on the upper and lower lobes of the left lung. Tissue samples were collected for culture, and the thoracic cavity was lavaged with antibiotic solution. Talc was used locally in the area for pleurodesis. Post-procedure, two 24-French Blake drains were placed, and an intercostal nerve block was performed for pain management. Following the surgical procedure, the patient was transferred to the surgical ward for postoperative care. Both chest tubes were set to a continuous suction at -20 mm Hg. Cultures of the pleural fluid showed no bacterial growth. On the third day after surgery, one of the chest tubes was removed. However, due to ongoing chest tube drainage and a lung that had yet to fully expand, the patient was discharged home on post-operative day 3 with a single remaining chest tube, which was connected to a Mini Atrium.

Outcome and follow-up

**Figure 6:**
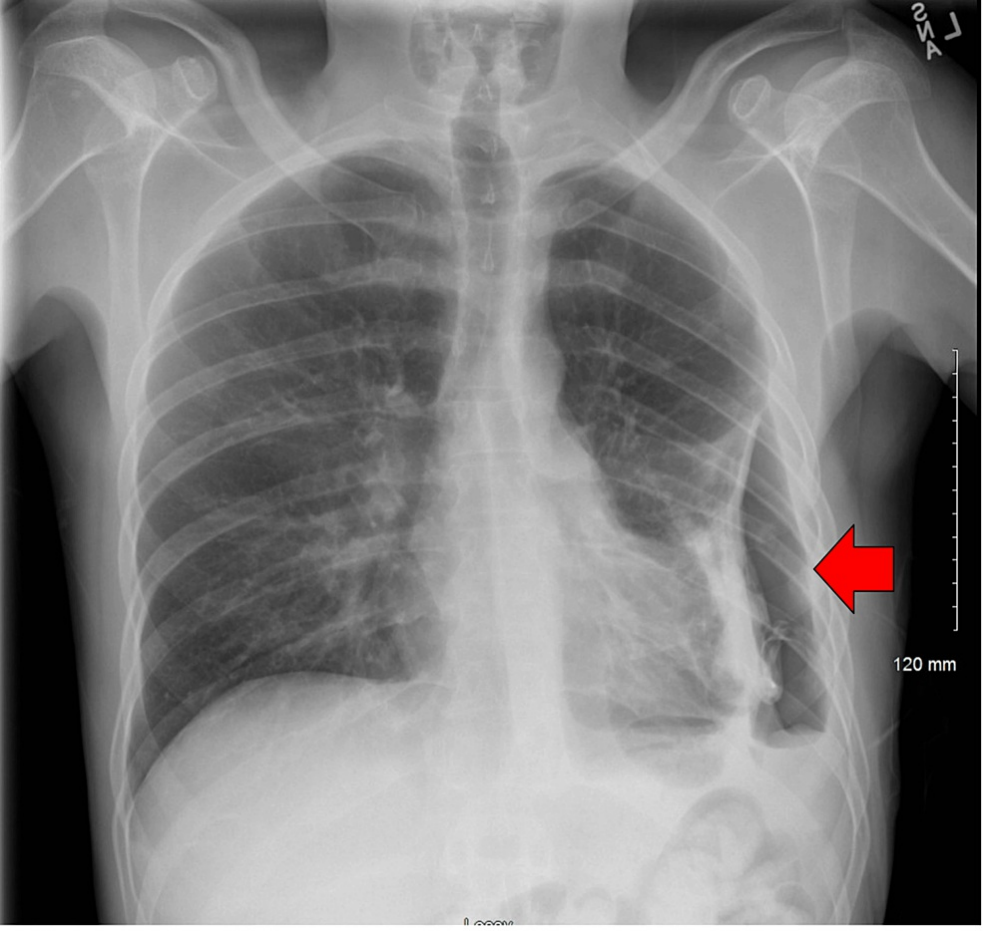
Chest X-ray three weeks post-discharge Chest X-ray showing stable left hydropneumothorax (red arrow) three weeks post-discharge.

Post-operative follow-up: The patient had follow-up appointments in the clinic at intervals of one week, two weeks, and three weeks post-discharge. Throughout this period, he reported feeling well, with no instances of shortness of breath either at rest or during physical activity. Since his discharge, the chest tube has been maintained on a water seal. The day before his most recent clinic visit, the chest tube was clamped to assess a persistent cavity in the base of his left lung, a condition noted in his discharge imaging. A chest X-ray confirmed that the cavity remained stable (Figure 6) compared to the initial post-discharge assessment. Given this stability, the decision was made to remove the chest tube, a procedure the patient underwent without any adverse change in his respiratory status.

## Discussion

The diagnosis and treatment of the 38-year-old patient with cerebral palsy (CP) in this case report brings to light several critical challenges and considerations in managing pneumothorax ex vacuo and trapped lung. This uncommon phenomenon is characterized by the introduction of air into the pleural space, a consequence of a vacuum-like negative intrapleural pressure stemming from lung collapse [2]. It is generally observed in patients experiencing bronchial obstructions due to various factors such as mucus plugs, aspiration of foreign bodies, and misplacement of the endotracheal tube [5-7].

The pathophysiology of pneumothorax ex vacuo, distinct from primary or secondary pneumothorax, does not involve a rupture in the visceral pleura [8]. Instead, it is marked by the formation of a thick fibrous peel due to neoplastic cell activity, entrapping the lung and preventing its re-expansion even after the effusion has been drained [6,8]. It is essential to note that the primary issue here is not the pleural effusion but the inability of the lung to expand, a condition initiated by lung collapse that induces negative pressure in the pleura, drawing fluid into the space [9].

Individuals with CP are more prone to respiratory complications, including empyema, due to factors such as aspiration risks, weakened immune systems, and frequent hospitalizations, which increase the risk of infections [9]. Moreover, mobility issues inherent to CP can lead to diminished lung function over time, thereby elevating the risk of respiratory infections, including empyema [9]. This necessitates a multidisciplinary approach involving various specialists to optimize patient outcomes, given the patient's pre-existing condition of CP, which complicates both diagnostic and treatment plans.

Despite initial indications of an infectious etiology based on laboratory findings, pleural fluid analysis did not confirm infection or malignancy, emphasizing the critical role of a multimodal diagnostic approach that encompasses imaging, laboratory studies, and clinical evaluations for precise diagnosis. The development of pneumothorax ex vacuo post drainage of pleural fluid posed a diagnostic dilemma.

The surgical intervention initially envisioned as a minimally invasive VATS had to be converted to a more invasive thoracotomy due to unforeseen pleural thickening, highlighting the need for adaptable surgical planning, especially when dealing with patients with complex clinical backgrounds or unpredictable pathology. The dense pleural adhesions and thickening encountered during surgery necessitated maximum decortication, albeit raising questions on how to foresee these challenges in preoperative planning and balancing decortication with the risk of damaging the underlying lung parenchyma.

Postoperative management mirrored a nuanced approach to patient care, including the placement of two 24-French Blake drains and the administration of an intercostal nerve block for pain management. The case serves as a potent illustration of how existing comorbidities can significantly influence the approach and management of even rare and specific conditions like pneumothorax ex vacuo and trapped lung, highlighting the benign nature of pneumothorax ex vacuo, which rarely enlarges or leads to tension pneumothorax, and emphasizing that patients remain asymptomatic, averting the need for chest tube treatment.

## Conclusions

This case report elucidates the diagnosis and management of pneumothorax ex vacuo in a patient with cerebral palsy, emphasizing the benign nature of this rare condition which arises from a vacuum-like negative intrapleural pressure due to lung collapse. Despite its concerning presentation, it generally does not necessitate chest tube treatment, as the primary issue is the unexpandable lung unresponsive to pleural drainage. The case advocates for a comprehensive diagnostic approach, combining imaging, laboratory studies, and clinical evaluations, and emphasizes the necessity for flexible surgical planning to anticipate unpredictable pathologies, especially in patients with pre-existing conditions that can complicate diagnostic and treatment paths. It also highlights the critical role of a multidisciplinary team in optimizing patient outcomes, particularly when managing individuals with cerebral palsy who are more susceptible to respiratory complications including empyema. The case ultimately serves as a reminder of the benign nature of pneumothorax ex vacuo, urging clinicians to approach it with a well-informed and cautious perspective.
